# Optimizing UniFrac with OpenACC Yields Greater Than One Thousand Times Speed Increase

**DOI:** 10.1128/msystems.00028-22

**Published:** 2022-05-31

**Authors:** Igor Sfiligoi, George Armstrong, Antonio Gonzalez, Daniel McDonald, Rob Knight

**Affiliations:** a San Diego Supercomputing Center, University of California, San Diego, La Jolla, California, USA; b Bioinformatics and Systems Biology Program, University of California, San Diego, La Jolla, California, USA; c Department of Pediatrics, University of California, San Diego, La Jolla, California, USA; d Center for Microbiome Innovation, University of California, San Diego, La Jolla, California, USA; e Department of Computer Science and Engineering, University of California, San Diego, La Jolla, California, USA; f Department of Bioengineering, University of California, San Diego, La Jolla, California, USA; University of Colorado Anschutz Medical Campus

**Keywords:** microbiome, GPU, OpenACC, optimization, UniFrac

## Abstract

UniFrac is an important tool in microbiome research that is used for phylogenetically comparing microbiome profiles to one another (beta diversity). Striped UniFrac recently added the ability to split the problem into many independent subproblems, exhibiting nearly linear scaling but suffering from memory contention. Here, we adapt UniFrac to graphics processing units using OpenACC, enabling greater than 1,000× computational improvement, and apply it to 307,237 samples, the largest 16S rRNA V4 uniformly preprocessed microbiome data set analyzed to date.

**IMPORTANCE** UniFrac is an important tool in microbiome research that is used for phylogenetically comparing microbiome profiles to one another. Here, we adapt UniFrac to operate on graphics processing units, enabling a 1,000× computational improvement. To highlight this advance, we perform what may be the largest microbiome analysis to date, applying UniFrac to 307,237 16S rRNA V4 microbiome samples preprocessed with Deblur. These scaling improvements turn UniFrac into a real-time tool for common data sets and unlock new research questions as more microbiome data are collected.

## OBSERVATION

The study of microbiomes has rapidly expanded over the past decade. One commonly used method in microbiome analyses is UniFrac (1), a phylogenetic measure of beta-diversity, which allows researchers to assess differences between pairs of microbiome profiles. UniFrac is particularly useful for microbial community analysis, because the distance computed accounts for the evolutionary relationships between microbes present within a sample. Other distance metrics, such as Euclidean distance, Bray-Curtis, and Jaccard, make the unrealistic implicit assumption that all organisms are equally related, which can lead to statistical artifacts (2, 3). These artifacts are worsened with sparse data matrices (4, 5), typical of real-world microbiome data sets, because most kinds of microbes are not found in most samples. UniFrac, being able to exploit phylogenetic relationships among features (e.g., 16S rRNA sequences, although it can be used on any data yielding a tree), can produce meaningful comparisons between samples even if they lack exact features in common (1).

**FIG 1:**
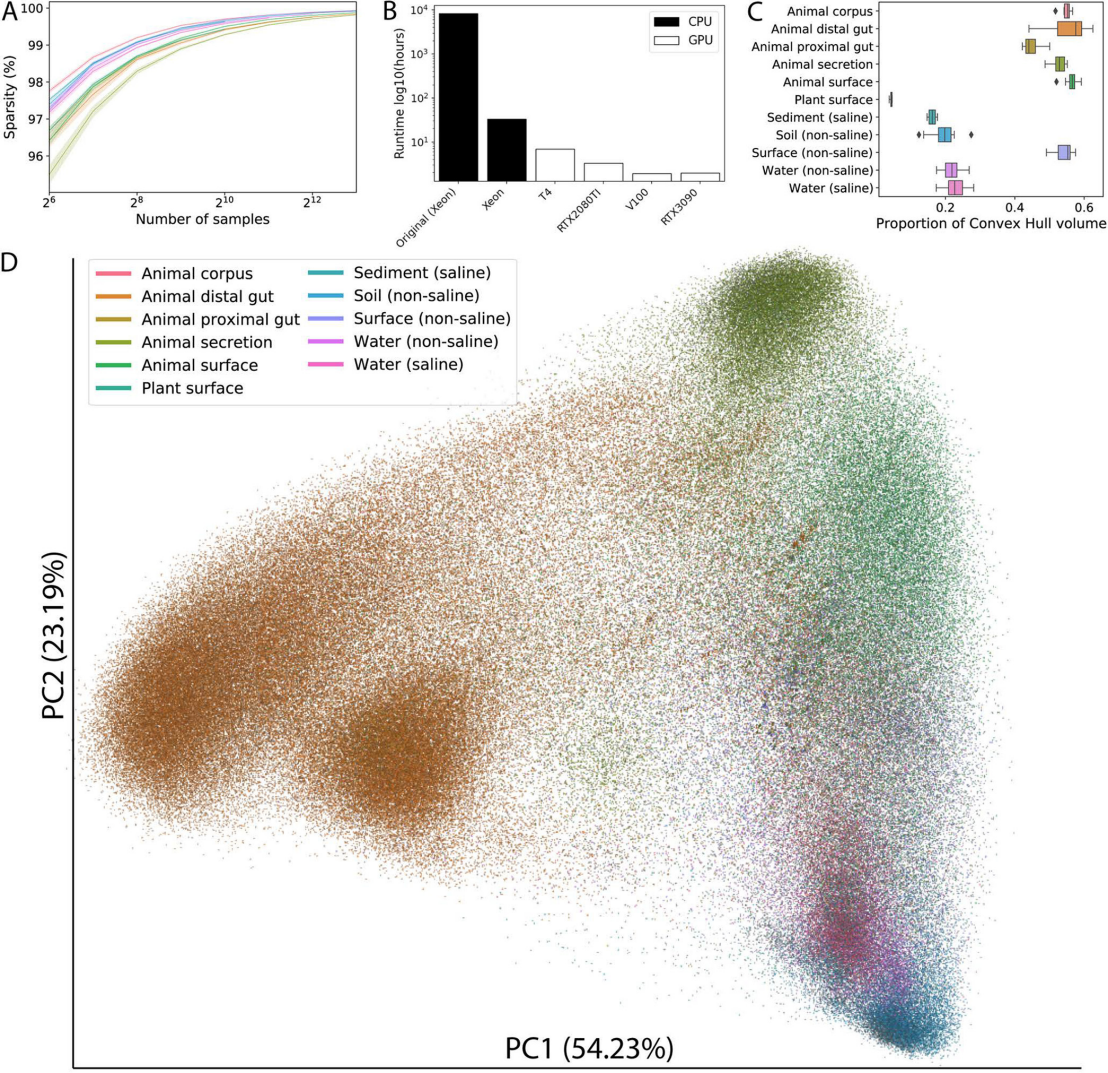
Optimized UniFrac. (A) Sparsity of microbiome data as a function of the number of samples stratified by environments with at least 1,000 samples (307k sample set), representing 92.37% of the total number of samples. (B) Runtime of optimized UniFrac using the 307k sample set. Black bars represent CPUs, and white bars show GPUs. (C) The proportion of the total convex hull volume from computed over principal coordinates for environments with at least 1,000 samples (307k sample set; volumes obtained by randomly selecting 1,000 samples from each environment 10 times and computing the convex hull volume over the first three principal coordinates for those samples, normalized to the total convex hull volume of all 307k samples). (D) A principal coordinates plot of the first two axes from 307k public and anonymized private 16S rRNA V4 samples from Qiita, colored by the Earth Microbiome Project Ontology level 3. An interactive version of the plot can be accessed at https://bit.ly/unifrac-pcoa-307k.

In recent years, microbiome studies have transitioned from experimental designs with a few hundred samples to designs with tens of thousands of samples. The sparsity of microbiome data tends to increase as a function of the number of samples under investigation, even within a single environment where the sparsity can be in excess of 99.8% (Fig. 1A). The use of larger sample size is important because, similar to human genomic studies, the amount of variation that exists within each environment type (6) can be high. By increasing sample size, researchers can detect subtle associations with the microbiome (7). Efforts under way right now, such as the Earth Microbiome Project (8) (EMP) and the American Gut/Microsetta Initiative (9), in part aim to provide large sample sizes crucial for untangling the many factors that influence microbial community composition, pushing the limits of existing tools as they operate on data sets beyond their original design considerations.

Having an efficient and scalable implementation for computing UniFrac thus is crucial for scaling individual microbiome studies and for large meta-analyses. Striped UniFrac was recently proposed and implemented, and it exhibits almost linear scaling with respect to the number of compute nodes used (10). However, the existing implementation does not scale linearly with the number of central processing unit (CPU) cores on a single node due to its memory-heavy nature. Massively parallel algorithms, especially memory-heavy ones, are natural candidates for computation on graphics processing unit (GPU) architectures. A key design factor was avoiding the generation of CPU-only and GPU-only code paths to reduce barriers for use. These constraints led to using OpenACC (11), which allows coexistence of CPU and GPU compute and conditional creation of GPU offload sections. OpenACC is supported by GCC and the NVIDIA HPC Toolkit (12), and it operates as a series of pragma directives to a compiler. The methodology used by OpenACC is similar to that of OpenMP (13) and spares the developer the manual device management typical in low-level frameworks like OpenCL while also avoiding the device-specific constraints of CUDA.

Porting to GPUs (14) and subsequent optimization (15) of the implementation underwent multiple phases. Expanded detail on each of these changes can be found in online methods and Fig. S1 and S2 in the supplemental material. The following steps were used. (i) Implementing a temporary unified memory buffer to support the memory access patterns of OpenACC. This fused inner loops and increased the available parallelism. (ii) Batching data to reduce the total number of GPU kernel invocations. (iii) Reorganizing loops and memory alignment to improve cache locality. (iv) Assessing floating point precision requirements and the support of 32-bit (single precision) floats. (v) Leveraging the sparse nature of the inputs within the UniFrac inner loops.

10.1128/msystems.00028-22.1 FIG S1 Examples of critical code changes. Dark blue highlights OpenACC constructs, while light blue shows specific changes. (A) The creation of a unified temporary buffer where time-consuming code could compute in and where the final result is stored. As part of this change, we switched to pointer manipulation for memory access. These changes fused loops and increased the available parallelism. (B) The batching strategy used to reduce the number of kernel invocations, allowing UniFrac inner loops to process data from many buffers at once. (C) Following the batching strategy, it became apparent that these buffers were being accessed multiple times within a single GPU kernel, resulting in poor cache utilization. A small reorganization of the main loop, and memory alignment, allowed for maximizing cache locality. These examples reflect changes shown at https://github.com/biocore/unifrac/blob/875a7b5925a6f0d5c3119a6fde6b01b7a40d7614/sucpp/unifrac_task.cpp#L104. Download FIG S1, TIF file, 1.4 MB. Copyright © 2022 Sfiligoi et al. 2022 Sfiligoi et al. https://creativecommons.org/licenses/by/4.0/ This content is distributed under the terms of the Creative Commons Attribution 4.0 International license.

10.1128/msystems.00028-22.2 FIG S2 Examples of code optimizations. Some constructs are artificially simplified with “….” (A) Parallel precalculation of zeros. (B) How the zero maps are leveraged. (C) Precalculation of bitmaps for unweighted UniFrac. (D) Example of how the lookups are performed. These examples reflect changes shown at https://github.com/biocore/unifrac-binaries/blob/d8c0ed6daab969fa3a3de30fe0b8c9c7a70704d2/src/unifrac_task.cpp#L644. Download FIG S2, TIF file, 1.3 MB. Copyright © 2022 Sfiligoi et al. 2022 Sfiligoi et al. https://creativecommons.org/licenses/by/4.0/ This content is distributed under the terms of the Creative Commons Attribution 4.0 International license.

Benchmarking of UniFrac was performed on three 16S rRNA V4 data sets: the previously described 27,751-sample set (EMP) from the Earth Microbiome Project (8), the previously described 113,721-public-sample set (113k) from Qiita (16) used in Striped UniFrac (10), and a 307,237-sample set (307k) composed of public and anonymized private samples from Qiita. The EMP and 113k sets were evaluated on multiple hardware targets, including consumer- and server-oriented products (Tables 1 and 2). Evaluation of the 307k sample set was limited to high-performance hardware, as the memory required is not typically found on consumer-grade products (Fig. 1B). All evaluations were performed on job-exclusive systems. Numerous hardware combinations yielded performance gains in excess of 1,000× relative to the original Striped UniFrac on the same hardware. Notably, the use of an NVIDIA RTX3090 in an Intel Xeon Gold 6140 host system resulted in a 4,200× reduction in walltime for unweighted UniFrac on the 307k set (Table 3), requiring just under 2 h of walltime.

**TABLE 1:** Speedups on the EMP data set relative to a few different architectures for unweighted UniFrac^*a*^

Platform	RAM (GB)	Runtime (min)	Speedup	GPU speedup	Mobile speedup
Original CPU Xeon Gold 6242	5.6	504	1×		
CPU Mobile i7-8565U	8.1	28.2	18×		1×
CPU Mobile i7-8850H	8.1	18.7	27×		1.5×
CPU Xeon Gold 6242	8.1	4.8	105×	1×	
GPU Mobile GTX 1050 Max-Q	6.6	3.8	170×	1.3×	7.4×
GPU T4	7.8	1.5	340×	3.2×	
GPU RTX2080TI	8.4	0.73	690×	6.6×	
GPU V100 PCIE 32GB	8.2	0.75	670×	6.4×	
GPU A100 PCIE 40GB	7.8	0.62	810×	7.7×	
GPU RTX3090	8.4	0.53	950×	9.0×	
GPU RTX8000	7.8	0.48	1,050×	10.0×	

a Speedup is relative to performance on the same data using Striped UniFrac from McDonald et al. (10). In all cases, all available compute resources for an architecture were utilized. Peak resident memory for the runs is provided.

**TABLE 2:** Speedups on the 113k data set relative to a few different architectures for unweighted UniFrac^*a*^

Platform	RAM (GB)	Runtime (h)	Speedup	GPU speedup	No. of chunks
Original CPU Xeon Gold 6242	5.5	498	1×		36
CPU Mobile i7-8850H	Not collected	10	50×		12
CPU Xeon Gold 6242	148	3	166×	1×	1
GPU Mobile GTX 1050 Max-Q	3.6	3	166×	1×	36
GPU T4	38	0.68	730×	4.4×	4
GPU RTX2080TI	27	0.32	1,560×	9.4×	6
GPU V100 PCIE 32GB	75	0.22	2,260×	13.6×	2
GPU RTX3090	51	0.19	2,600×	15.8×	3

a Speedup is relative to performance on the same data using Striped UniFrac from McDonald et al. (10). In all cases, all available compute resources for an architecture were utilized. Peak resident memory for the runs is provided; however, the amount of maximum memory used for processing is a function of how many chunks are processed at one time. The largest memory use comes from creating the distance matrix that is *N*^2^ to the number of samples (not shown) and is effectively invariant to the architecture.

**TABLE 3:** Speedups on the 307k data set relative to a few different architectures for unweighted UniFrac^*a*^

Platform	RAM (GB)	Runtime (h)	Speedup	GPU speedup	No. of chunks
Original CPU Xeon Gold 6242	7	8,326	1×		482
CPU Xeon Gold 6242	184	33.1	252×	1x	6
GPU T4	38	6.9	1,200×	4.8×	30
GPU RTX2080TI	33	3.3	2,530×	10x	36
GPU V100 PCIE 32GB	85	1.93	4,300×	17.2×	13
GPU RTX3090	47	1.97	4,200×	16.8×	24

a Speedup is relative to performance on the same data using Striped UniFrac from McDonald et al. (10). In all cases, all available compute resources for an architecture were utilized. Peak resident memory for the runs is provided; however, the amount of maximum memory used for processing is a function of how many chunks are processed at one time. The largest memory use comes from creating the distance matrix that is *N*^2^ to the number of samples (not shown) and is effectively invariant to the architecture.

To reduce serialization and deserialization expense when interacting with these distance matrices, we implemented an HDF5-based (17) binary distance matrix type for memory-mapped random access to sample distances. We additionally implemented a CPU-based principal coordinate analysis directly into the UniFrac codebase such that these coordinates are automatically computed at runtime (adds 4 min to runtime on the 307k sample set), storing the coordinate details in the same HDF5 container. A Python API wraps the optimized code using Cython (18) and provides programmatic interaction with these types, including easily representing the coordinates in the scikit-bio (19) OrdinationResults object, which is compatible with QIIME 2 (20). Convex hull volumes computed over the first three principal coordinates of the 307k sample set reveal that animal-associated environments tend to occupy a larger portion of the space (Fig. 1C). The first two coordinates visualized relative to the Earth Microbiome Project Ontology level 3 emphasize the dramatic difference in host-associated versus environmental microbiome samples (Fig. 1D; an interactive version of the plot can be found at https://bit.ly/unifrac-pcoa-307k).

Having access to effective but also fast computational methods for microbiome analysis is essential. UniFrac has long been an important tool in microbiome research, and our work now allows many analyses that were previously relegated to large compute clusters to be performed with much lower resource requirements. Even the largest data sets currently envisaged could be processed in reasonable time with a server-class GPU or cloud-based GPUs, while smaller but still massive data sets like the EMP now can be processed on laptops.

In addition to the substantial reductions in runtime for UniFrac, we also show why OpenACC should be considered for other performance-critical C/C++ code within and beyond the microbiome field. It allows for a single codebase for both CPU and GPU code, an important consideration for reducing long-term support burden, and is similar in many respects to the popular OpenMP framework. However, the experience emphasizes the importance of budgeting for software developers and specialists. Many codebases that support the microbiome field are written in R or Python due to their accessibility, which tend to obscure low-level aspects of the hardware necessary for fine-tuned optimization. Rewriting complex algorithms in low-level C/C++ creates opportunities to maximize hardware performance but incurs a challenging engineering effort. Relatively few code changes to the C/C++ code were necessary to obtain large performance gains, suggesting further exploration of OpenACC should be considered for memory-intensive C/C++ code in the microbiome space. We hope the work here is viewed as an example of the dramatic gains available to bioinformatic researchers with the adoption of high-performance frameworks such as OpenACC and that UniFrac acts as an exemplar for the gains possible. We encourage researchers in the bioinformatic community to examine OpenACC for use in their tools to accommodate the incredible scaling challenges faced by the field with the ever-increasing volumes of data being generated.

### Porting and optimizing UniFrac.

The original Striped UniFrac implementation is composed of a set of tight loops that operate on adjacent, independent memory cells and a set of memory buffers. We set out to adapt the implementation to use GPUs with OpenACC, as it simplifies device and memory management and the utilization of heterogeneous computational targets. OpenACC does not allow for passing an array of pointers into OpenACC sections. To minimize the refactoring complexity, we first created a unified temporary buffer where time-consuming code could operate and that holds a final copy at the end of the computation. With a unified memory buffer in place, we then switched the code to use pointer manipulation to access the necessary memory cells. This change fused loops, increasing the available parallelism. A comparison of a subset of the code before and after is provided in Fig. S1a. This relatively light change was all that was needed to compile a working version of UniFrac that could run on a GPU with appreciable performance gains: the EMP could be computed in 92 min on an NVIDIA Tesla V100 GPU, representing over 5× gain over the original implementation using a 16-core Intel Xeon Gold 6242 CPU.

A GPU port typically requires further optimization to saturate the available computational resources. Code profiling was performed, leading to the identification of hot spots within the inner loops that perform the UniFrac calculations among the different UniFrac variants. A major bottleneck was observed related to repeated writes to the main memory buffer residing with the CPU, such that each GPU kernel invocation would access the CPU buffer, transform the information, and write back to the updated CPU buffer sequentially. Writing to memory takes substantially more time than reading from it, and each input/output operation against main memory from a GPU kernel carries overhead. This observation suggested a batching strategy where many input buffers were provided to a single kernel invocation, allowing the UniFrac inner loops to process data from many input buffers at once before writing to the CPU memory buffer. This buffering increased the memory footprint of the application by 1% but resulted in a further reduction in runtime on the EMP data set to 33 min on an NVIDIA Tesla V100 GPU. The updated code snippet is available in Fig. S1b. By grouping the input buffers, it became obvious that these buffers were being accessed multiple times within a single GPU kernel; the access pattern was such that data reuse was deferred, resulting in poor cache utilization. A small reorganization of the main loop, and memory alignment, allowed for maximizing cache locality (Fig. S1c) and further reducing EMP compute time to 12 min, representing a roughly 40× improvement over the original implementation. Excitingly, with OpenACC disabled, we observed a 4× reduction in runtime with the EMP on CPUs, indicating the implementation optimization was generally beneficial.

We next explored whether the use of single (fp32) versus double (fp64) precision floating point values had a difference in results. Consumer-grade GPUs, such as those found in laptops, are not optimized for fp64 operations. This is largely due to there being negligible benefit for graphics and physics models in the popular video games that drive the market. UniFrac was originally implemented using fp64, but it was not obvious whether the increased precision yielded different results. To test this, we compiled UniFrac using fp32 and observed a nearly identical result with the EMP-to-fp64 distances (Mantel *R*^2^, 0.99999; *P* < 0.001), with a further 2× to 6× reduction in runtime. As such, we recommend the fp32-enabled code for most microbiome discovery work, particularly if on consumer equipment, and that users should rely on the fp64 variant only in the unusual situation where the relative abundances of the input data or the tree branch lengths exhibit a very high dynamic range in elements of the distance matrix that contribute substantially to downstream results, e.g., after dimensionality reduction.

As a further step, we considered the sparsity of the inputs to UniFrac. In previous algorithms of UniFrac, including Striped UniFrac and Fast UniFrac, the data at the point of UniFrac calculation are expressed as dense vectors. This is true even if the input data are a sparse matrix. For Striped UniFrac, the use of dense vectors did not require substantial memory, as the dense vector representations were only used on an as-needed basis. However, this resulted in a large amount of compute within the UniFrac kernels being expended on zeros. By retaining proportions as sparse vectors and redesigning the inner loops to be “sparse-aware” (Fig. S2A and B), the total compute per tree vertex was reduced by an average of 90%, resulting in drastic speedup of the GPU code. A single CPU thread now was not enough to feed the data fast enough to the GPU, so the dense vector generation code was made multithreaded through the use of OpenMP. The EMP compute time using fp32 math thus was reduced to 2.4 min on an NVIDIA V100 GPU, roughly a 200× improvement over the original implementation. As expected, with OpenACC disabled, we observed a significant speedup on CPUs too, resulting in an EMP compute of less than 14 min on the Intel Xeon Gold 6242 CPU. UniFrac compute on larger samples resulted in even more significant time reductions on the NVIDIA V100 GPU, namely, 750× for the 113k data set and 1,500× for the 307k data set.

For unweighted UniFrac, which is qualitative, meaning the metric operates on presence/absence data, we were further able to reduce the overhead by utilizing bit vectors and bitwise operations. Furthermore, a lookup table has been introduced as a memory/compute trade-off, further reducing the runtime (Fig. S2C and D). Together with sparsity considerations, the EMP compute time was reduced to a mere 44 s on an NVIDIA V100 GPU for an approximately 650× speedup. With OpenACC disabled, the speedup on CPUs is approximately 100×. As before, the unweighted UniFrac speedup increases with sample size; using an NVIDIA V100 PCIE 32GB GPU with a Xeon Silver 4110, we measure a 2,200× speedup for the 113k data set and a 4,300× speedup for the 307k data set.

### Benchmark data sets.

Benchmarking surrounded three data sets. The first was the published Earth Microbiome Project (8) (EMP), representing 27,751 samples. The second data set, here referred to as 113k, corresponds to the public portion of Qiita (16) as of February 2017. Details of data set construction and treatment for the EMP and 113k set are described in Striped UniFrac documentation (10). The third data set corresponds to the entirety of the public and anonymized private portions of Qiita from the 90-nucleotide Deblur 16S rRNA V4 context, obtained from redbiom (21) in July 2020. Briefly, 326,926 samples were obtained from Qiita, and features were inserted into Greengenes 13_8 99% (22) using SEPP (23). Features that failed insertion were removed from the samples, followed by rarefying the samples to 500 sequences per sample. We opted for a rarefaction level of 500 sequences per sample to ensure a computationally difficult problem was presented, as the number of samples is the dominant scaling factor for UniFrac (see the supplemental material of reference 10 for detailed discussion on scaling). Following rarefaction, the final feature table contained 307,237 samples and 1,264,796 features with a sparsity of 99.994%.

### Benchmarking detail.

All benchmarking was performed on job-exclusive systems, including systems hosted by the Pacific Research Platform (24). Timings were obtained using GNU Time.

### Sparsity calculation.

Sets of samples, per EMPO type, were randomly pulled from the 307k table at increasing powers of 2, in steps from 64 to 8,192, with 10 iterations at each step, followed by computing the number of zero elements relative to the total number of elements a dense representation of the feature table would contain. Only those environments with at least 1,000 samples were plotted.

### Convex hull calculation.

Convex hull volumes were computed from the first three principal coordinates using SciPy v1.5.2 (25). Specifically, the total volume was computed for all samples. Next, for each environment with at least 1,000 samples, a random subset of 1,000 samples was pulled 10 times (with replacement). The volume of the sample subset was computed and normalized with respect to the total volume for all samples.

### Code availability.

An implementation of UniFrac is available on GitHub (https://github.com/biocore/unifrac-binaries) with a Python wrapper (https://github.com/biocore/unifrac); both repositories use a BSD-3 license. The specific version of the source code used here is available from Zenodo under no. 10.5281/zenodo.6127564. A CPU- and GPU-optimized build can be installed using conda from Bioconda channel (26) and is part of QIIME 2’s q2-diversity plugin. All builds provide a command-line interface, a C shared library, and a Python application program interface. Most variants of UniFrac are implemented, including unweighted, weighted, generalized, and variance-adjusted UniFrac (1, 27–29). An accessioned tutorial companion showing how to download the data used here, install UniFrac, operate it in GPU or CPU mode, and generate figures, all from Google Colabs, is available from Zenodo under no. 10.5281/zenodo.6127558. The benchmarking scripts and specifics are available from Zenodo under no. 10.5281/zenodo.6127654.

### Data availability.

The data sets analyzed within the current study are part of the Qiita repository and were extracted from an internal redbiom cache that indexes public and private study data. A BIOM table, unweighted UniFrac distance matrix, principal coordinates, and limited sample metadata representing the 307k data set is available from Zenodo under no. 10.5281/zenodo.6127601. Studies noted as private by study owners were included; however, the corresponding study and sample identifiers have been anonymized.

## References

[1] Lozupone C, Knight R. 2005. UniFrac: a new phylogenetic method for comparing microbial communities. Appl Environ Microbiol 71:8228–8235. doi:10.1128/AEM.71.12.8228-8235.2005. 16332807 PMC1317376

[2] Hamady M, Knight R. 2009. Microbial community profiling for human microbiome projects: tools, techniques, and challenges. Genome Res 19:1141–1152. doi:10.1101/gr.085464.108. 19383763 PMC3776646

[3] Vázquez-Baeza Y, Gonzalez A, Smarr L, McDonald D, Morton JT, Navas-Molina JA, Knight R. 2017. Bringing the dynamic microbiome to life with animations. Cell Host Microbe 21:7–10. doi:10.1016/j.chom.2016.12.009. 28081445

[4] Morton JT, Toran L, Edlund A, Metcalf JL, Lauber C, Knight R. 2017. Uncovering the horseshoe effect in microbial analyses. mSystems 2:e00166-16. doi:10.1128/mSystems.00166-16. 28251186 PMC5320001

[5] Armstrong GW, Rahman G, Martino C, McDonald D, Gonzalez A, Mishne G, Knight R. 2022. Applications and comparison of dimensionality reduction methods for microbiome data. Front Bioinform doi:10.3389/fbinf.2022.821861. PMC9580878 36304280

[6] Allaband C, McDonald D, Vázquez-Baeza Y, Minich JJ, Tripathi A, Brenner DA, Loomba R, Smarr L, Sandborn WJ, Schnabl B, Dorrestein P, Zarrinpar A, Knight R. 2019. Microbiome 101: studying, analyzing, and interpreting gut microbiome data for clinicians. Clin Gastroenterol Hepatol 17:218–230. doi:10.1016/j.cgh.2018.09.017. 30240894 PMC6391518

[7] Vujkovic-Cvijin I, Sklar J, Jiang L, Natarajan L, Knight R, Belkaid Y. 2020. Host variables confound gut microbiota studies of human disease. Nature 587:448–454. doi:10.1038/s41586-020-2881-9. 33149306 PMC7677204

[8] Thompson LR, Sanders JG, McDonald D, Amir A, Ladau J, Locey KJ, Prill RJ, Tripathi A, Gibbons SM, Ackermann G, Navas-Molina JA, Janssen S, Kopylova E, Vázquez-Baeza Y, González A, Morton JT, Mirarab S, Zech Xu Z, Jiang L, Haroon MF, Kanbar J, Zhu Q, Jin Song S, Kosciolek T, Bokulich NA, Lefler J, Brislawn CJ, Humphrey G, Owens SM, Hampton-Marcell J, Berg-Lyons D, McKenzie V, Fierer N, Fuhrman JA, Clauset A, Stevens RL, Shade A, Pollard KS, Goodwin KD, Jansson JK, Gilbert JA, Knight R, Earth Microbiome Project Consortium. 2017. A communal catalogue reveals Earth’s multiscale microbial diversity. Nature 551:457–463. doi:10.1038/nature24621. 29088705 PMC6192678

[9] McDonald D, Hyde E, Debelius JW, Morton JT, Gonzalez A, Ackermann G, Aksenov AA, Behsaz B, Brennan C, Chen Y, DeRight Goldasich L, Dorrestein PC, Dunn RR, Fahimipour AK, Gaffney J, Gilbert JA, Gogul G, Green JL, Hugenholtz P, Humphrey G, Huttenhower C, Jackson MA, Janssen S, Jeste DV, Jiang L, Kelley ST, Knights D, Kosciolek T, Ladau J, Leach J, Marotz C, Meleshko D, Melnik AV, Metcalf JL, Mohimani H, Montassier E, Navas-Molina J, Nguyen TT, Peddada S, Pevzner P, Pollard KS, Rahnavard G, Robbins-Pianka A, Sangwan N, Shorenstein J, Smarr L, Song SJ, Spector T, Swafford AD, Thackray VG, The American Gut Consortium, Knight R. 2018. American gut: an open platform for citizen science microbiome research. mSystems 3:e00031-18. doi:10.1128/mSystems.00031-18. 29795809 PMC5954204

[10] McDonald D, Vázquez-Baeza Y, Koslicki D, McClelland J, Reeve N, Xu Z, Gonzalez A, Knight R. 2018. Striped UniFrac: enabling microbiome analysis at unprecedented scale. Nat Methods 15:847–848. doi:10.1038/s41592-018-0187-8. 30377368 PMC7250580

[11] Wienke S, Springer P, Terboven C, van Mey D. 2012. OpenACC—first experiences with real-world applications, p 859–870. In Euro-Par 2012 parallel processing. Springer, Berlin, Germany.

[12] NVIDIA. 2020. HPC SDK. https://developer.nvidia.com/hpc-sdk.

[13] Dagum L, Menon R. 1998. OpenMP: an industry standard API for shared-memory programming. IEEE Comput Sci Eng 5:46–55. doi:10.1109/99.660313.

[14] Sfiligoi I, McDonald D, Knight R. 2020. Porting and optimizing UniFrac for GPUs, p 500–504. *In* Practice and experience in advanced research computing. Association for Computing Machinery, New York, NY. doi:10.1145/3311790.3399614.

[15] Sfiligoi I, McDonald D, Knight R. 2021. Enabling microbiome research on personal devices. *In* 2021 IEEE 17th International Conference on eScience. IEEE, Piscataway, NJ. doi:10.1109/eScience51609.2021.00035.

[16] Gonzalez A, Navas-Molina JA, Kosciolek T, McDonald D, Vázquez-Baeza Y, Ackermann G, DeReus J, Janssen S, Swafford AD, Orchanian SB, Sanders JG, Shorenstein J, Holste H, Petrus S, Robbins-Pianka A, Brislawn CJ, Wang M, Rideout JR, Bolyen E, Dillon M, Caporaso JG, Dorrestein PC, Knight R. 2018. Qiita: rapid, web-enabled microbiome meta-analysis. Nat Methods 15:796–798. doi:10.1038/s41592-018-0141-9. 30275573 PMC6235622

[17] HDF Group. 2022. The HDF5 library and file format. http://www.hdfgroup.org/HDF5.

[18] Behnel S, Bradshaw R, Citro C, Dalcin L, Seljebotn DS, Smith K. 2011. Cython: the best of both worlds. Comput Sci Eng 13:31–39. doi:10.1109/MCSE.2010.118.

[19] Rideout JR, et al. 2018. biocore/scikit-bio: scikit-bio 0.5.5: more compositional methods added. doi:10.5281/zenodo.2254379.

[20] Bolyen E, Rideout JR, Dillon MR, Bokulich NA, Abnet CC, Al-Ghalith GA, Alexander H, Alm EJ, Arumugam M, Asnicar F, Bai Y, Bisanz JE, Bittinger K, Brejnrod A, Brislawn CJ, Brown CT, Callahan BJ, Caraballo-Rodríguez AM, Chase J, Cope EK, Da Silva R, Diener C, Dorrestein PC, Douglas GM, Durall DM, Duvallet C, Edwardson CF, Ernst M, Estaki M, Fouquier J, Gauglitz JM, Gibbons SM, Gibson DL, Gonzalez A, Gorlick K, Guo J, Hillmann B, Holmes S, Holste H, Huttenhower C, Huttley GA, Janssen S, Jarmusch AK, Jiang L, Kaehler BD, Kang KB, Keefe CR, Keim P, Kelley ST, Knights D, et al. 2019. Reproducible, interactive, scalable and extensible microbiome data science using QIIME 2. Nat Biotechnol 37:852–857. doi:10.1038/s41587-019-0209-9. 31341288 PMC7015180

[21] McDonald D, Kaehler B, Gonzalez A, DeReus J, Ackermann G, Marotz C, Huttley G, Knight R. 2019. redbiom: a rapid sample discovery and feature characterization system. mSystems 4:e00215-19. doi:10.1128/mSystems.00215-19. 31239397 PMC6593222

[22] McDonald D, Price MN, Goodrich J, Nawrocki EP, DeSantis TZ, Probst A, Andersen GL, Knight R, Hugenholtz P. 2012. An improved Greengenes taxonomy with explicit ranks for ecological and evolutionary analyses of bacteria and archaea. ISME J 6:610–618. doi:10.1038/ismej.2011.139. 22134646 PMC3280142

[23] Janssen S, McDonald D, Gonzalez A, Navas-Molina JA, Jiang L, Xu ZZ, Winker K, Kado DM, Orwoll E, Manary M, Mirarab S, Knight R. 2018. Phylogenetic placement of exact amplicon sequences improves associations with clinical information. mSystems 3:e00021-18. doi:10.1128/mSystems.00021-18. 29719869 PMC5904434

[24] Smarr L, et al. 2018. The Pacific research platform: making high-speed networking a reality for the scientist, p 1–8. *In* Proceedings of the Practice and Experience on Advanced Research Computing. Association for Computing Machinery, New York, NY.

[25] Virtanen P, Gommers R, Oliphant TE, Haberland M, Reddy T, Cournapeau D, Burovski E, Peterson P, Weckesser W, Bright J, van der Walt SJ, Brett M, Wilson J, Millman KJ, Mayorov N, Nelson ARJ, Jones E, Kern R, Larson E, Carey CJ, Polat İ, Feng Y, Moore EW, VanderPlas J, Laxalde D, Perktold J, Cimrman R, Henriksen I, Quintero EA, Harris CR, Archibald AM, Ribeiro AH, Pedregosa F, van Mulbregt P, SciPy 1.0 Contributors. 2020. SciPy 1.0: fundamental algorithms for scientific computing in Python. Nat Methods 17:261–272. doi:10.1038/s41592-019-0686-2. 32015543 PMC7056644

[26] Grüning B, Dale R, Sjödin A, Chapman BA, Rowe J, Tomkins-Tinch CH, Valieris R, Köster J, Bioconda Team. 2018. Bioconda: sustainable and comprehensive software distribution for the life sciences. Nat Methods 15:475–476. doi:10.1038/s41592-018-0046-7. 29967506 PMC11070151

[27] Lozupone CA, Hamady M, Kelley ST, Knight R. 2007. Quantitative and qualitative beta diversity measures lead to different insights into factors that structure microbial communities. Appl Environ Microbiol 73:1576–1585. doi:10.1128/AEM.01996-06. 17220268 PMC1828774

[28] Chen J, Bittinger K, Charlson ES, Hoffmann C, Lewis J, Wu GD, Collman RG, Bushman FD, Li H. 2012. Associating microbiome composition with environmental covariates using generalized UniFrac distances. Bioinformatics 28:2106–2113. doi:10.1093/bioinformatics/bts342. 22711789 PMC3413390

[29] Chang Q, Luan Y, Sun F. 2011. Variance adjusted weighted UniFrac: a powerful beta diversity measure for comparing communities based on phylogeny. BMC Bioinformatics 12:118. doi:10.1186/1471-2105-12-118. 21518444 PMC3108311

