# Supplementary figures and images for: Optimizing UniFrac with OpenACC Yields Greater Than One Thousand Times Speed Increase
Source: mSystems. 2022 May 31;7(3):e00028-22. doi: 10.1128/msystems.00028-22 (PMC9239203; doi:10.1128/msystems.00028-22)

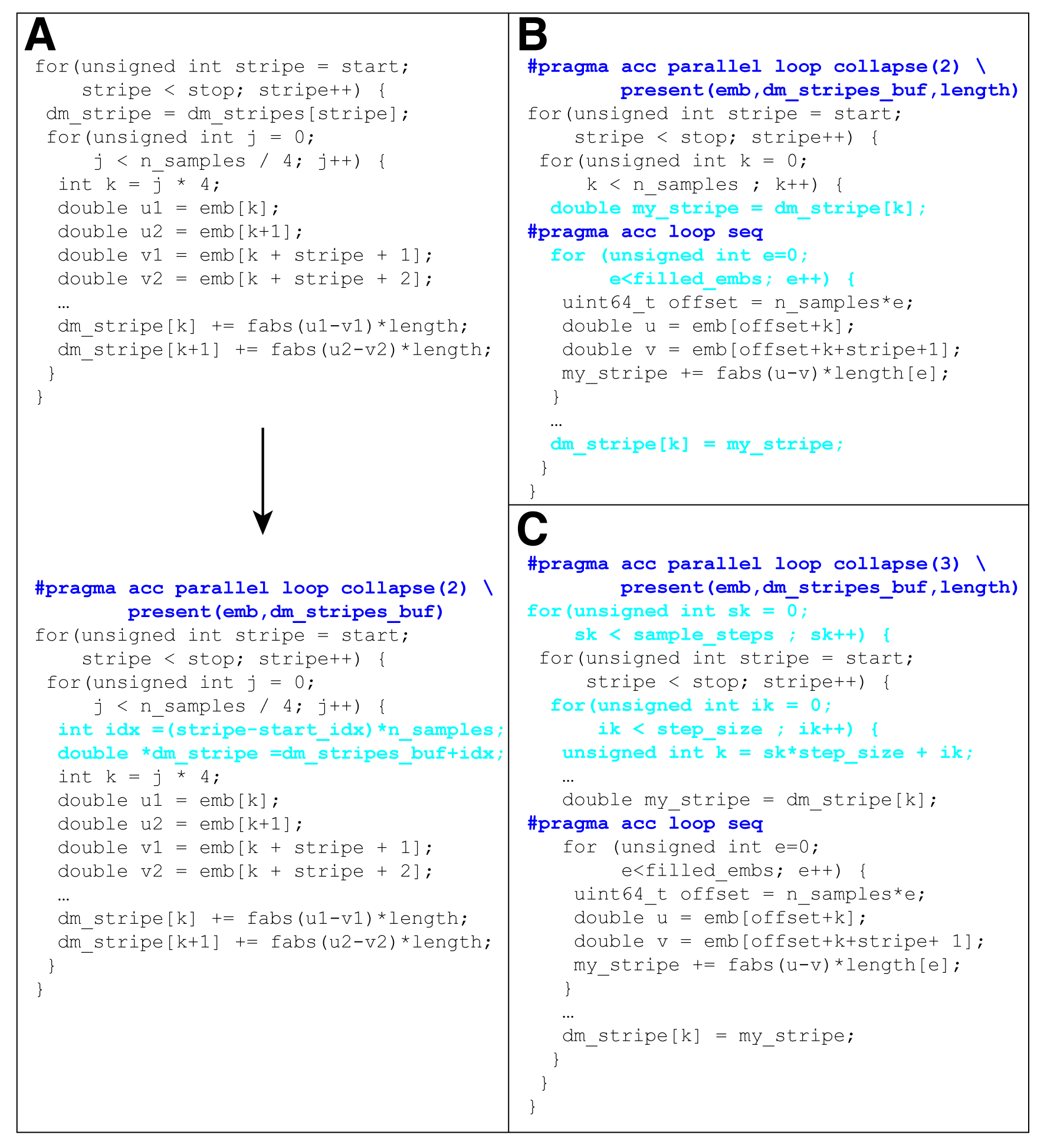

Supplement: FIG S1 [file msystems.00028-22-s0001.tif]

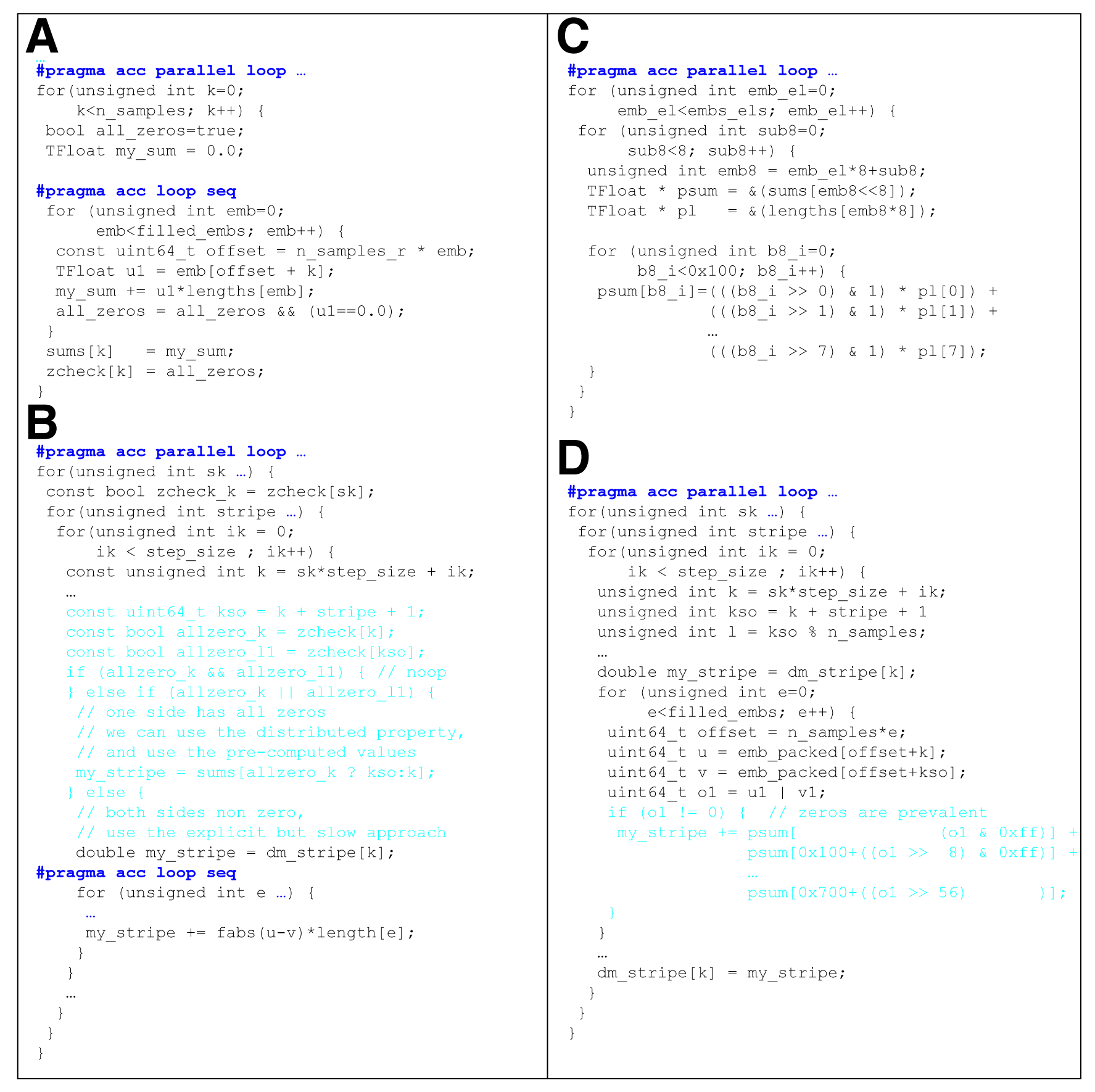

Supplement: FIG S2 [file msystems.00028-22-s0002.tif]
